# Supplementary material for: Nanopore Technology Applied to Targeted Detection of Tomato Brown Rugose Fruit Virus Allows Sequencing of Related Viruses and the Diagnosis of Mixed Infections
Source: Plants (Basel). 2023 Feb 22;12(5):999. doi: 10.3390/plants12050999 (PMC10005216; doi:10.3390/plants12050999)
Supplement: Supplementary file 1 [file plants-12-00999-s001.zip › plants-2203162-Figure S2.pdf]

```
Alignment of Sequence_1:  [ToBRFV isolate Tom-BA21 comp + RP MinIon primrs.xdna]
with  Sequence_2: [ToMMV isolate MX5 [NC_022230.1] .xdna]

Similarity : 5037/6398 (78,73 %)

Seq_1  1      GTGTATTTTTTACAACATA---TACCAACAACAACAAACAACAAACAACAACATCACAAT  57
                |||||| |||  |||     |||||||||||||||  |||||  |  |   |     |
Seq_2  1      --GTATTTATTATTACAACAATTACCAACAACAACAACAAACAACAACAACATTACATTT  58


Seq_1  58     TACTATTTACAACTACAATGGCATACACACAGACAGCTACCACATCCGCTTTGCTCGACA  117
              ||| || ||||||||||||||||||||||||| ||||||||  | |||||||||||| ||||
Seq_2  59     TACAATCTACAACTACAATGGCATACACACAAACAGCTACATCTTCCGCTTTGCTTGACA  118


Seq_1  118    CTGTCCGAGGTAACAATACCTTGGTCAACGATCTTGCGAAGCGGCGTCTTTATGACACAG  177
              ||||||| |||||||| ||| ||||||| |||||||| ||||| ||||||||||||||||
Seq_2  119    CTGTCCGGGGTAACAACACCCTGGTCAATGATCTTGCAAAGCGTCGTCTTTATGACACAG  178


Seq_1  178    CGGTCGACGAGTTCAACGCTCGTGATCGCAGGCCCAAAGTAAATTTTTCCAAAGTAATAA  237
              ||||||| || || |||||| | || |||||||||||||| || ||||| |||||| |||
Seq_2  179    CGGTCGATGAATTTAACGCTAGGGACCGCAGGCCCAAAGTCAACTTTTCTAAAGTAGTAA  238


Seq_1  238    GTGAGGAACAGACGCTTATTGCTACTAGGGCATATCCAGAATTCCAGATAACCTTCTATA  297
              ||||||||||||| |||||||||||  |||||||||| ||||||||||| || |||||||
Seq_2  239    GTGAGGAACAGACACTTATTGCTACCCGGGCATATCCTGAATTCCAGATCACGTTCTATA  298


Seq_1  298    ATACGCAGAACGCCGTGCATTCGCTTGCCGGTGGACTACGATCCTTAGAACTGGAATATC  357
              | || |||||||| || || |||||||| ||||| || ||||| ||||| ||||||||||
Seq_2  299    ACACTCAGAACGCAGTTCACTCGCTTGCGGGTGGTCTGCGATCATTAGAGCTGGAATATC  358


Seq_1  358    TAATGATGCAGATCCCGTACGGATCACTCACATATGATATAGGTGGGAATTTTGCATCTC  417
              | |||||||| ||||||||||| ||||| || ||||||||||| |||||||||||||| |
Seq_2  359    TGATGATGCAAATCCCGTACGGCTCACTGACGTATGATATAGGAGGGAATTTTGCATCAC  418


Seq_1  418    ATCTGTTCAAAGGACGGGCATATGTTCACTGCTGTATGCCCAATCTTGATGTCCGCGACA  477
              ||||||||||||| || ||||| ||||||||||||||||| ||||| |||||||||||| |
Seq_2  419    ATCTGTTCAAAGGGCGAGCATACGTTCACTGCTGTATGCCGAATCTGGATGTCCGCGATA  478


Seq_1  478    TAATGCGGCACGAAGGCCAGAAAGACAGTATAGAATTATACCTTTCCAGGCTTGAGCGGG  537
              | ||||||||||||||||| || || ||  | ||  |||||||  | ||||| ||  |||
Seq_2  479    TTATGCGGCACGAAGGCCAAAAGGATAGCGTCGAGCTATACCTCGCTAGGCTAGAAAGGG  538


Seq_1  538    GCAACAAAGTTGTCCCAAATTTCCAAAAGGAAGCTTTTGACAGATACGCTGAAACGCCAG  597
              |||||||  ||||||| ||  |||||||||||||| |||||||||||||||||||||||||
Seq_2  539    GCAACAAGTTTGTCCCGAACTTCCAAAAGGAAGCCTTTGACAGATACGCTGAAACGCCAG  598


Seq_1  598    ACGAAGTTGTCTGTCACAGTACCTTCCAAACGTGTACGCACCAGCAGGTGAAAAACACAG  657
              | ||||| |||||||||  ||||||||||||| |||| ||||  ||| | ||   ||||||
Seq_2  599    ATGAAGTAGTCTGTCACGATACCTTCCAAACCTGTAGGCACTCGCAAGAGATGTACACAG  658


Seq_1  658    GCAGGGTGTATGCTATTGCATTGCACAGTATATACGATATACCTGCTGATGAATTCGGAG  717
              | || ||||||||||||||  |||| || ||||||||| ||||| || ||||| ||||| |
Seq_2  659    GGAGAGTGTATGCTATTGCTCTGCATAGCATATACGACATACCAGCCGATGAGTTCGGCG  718


Seq_1  718    CGGCACTTTTAAGGAAAAATGTCCATGTTTGTTACGCCGCCTTCCACTTTTCCGAGAATT  777
              ||||| |  | || || ||||| ||||| || || ||||| |||||||| ||||||||||
Seq_2  719    CGGCATTACTTAGAAAGAATGTACATGTGTGCTATGCCGCTTTCCACTTCTCCGAGAATT  778


Seq_1  778    TACTTCTCGAAGATTCACACGTCAACCTTGACGAAATCAACGCGTGTTTTTCGCGTGATG  837
```

```
              |||||||||||||||| |||||||| || || ||||||||||| ||||||  | | ||||
Seq_2  779    TACTTCTCGAAGATTCGCACGTCAATCTCGATGAAATCAACGCATGTTTTCAGAGAGATG  838

Seq_1  838    GAGACAAGCTGACTTTTTCTTTCGCATCTGAGAGCACTTTAAATTATTGTCATAGTTATT  897
              | || | | | |||||||| || || |||||||| ||| | ||||||   ||||||| |
Seq_2  839    GCGATAGGTTAACTTTTTCCTTTGCTTCTGAGAGTACTCTTAATTATACGCATAGTTTTC  898

Seq_1  898    CTAATATTTTAAAATACGTGTGCAAAACTTACTTCCCGGCATCTAATAGAGAGGTCTACA  957
              ||||||||||||| || |||||||||||||||||||| || |||||||||||||| ||||
Seq_2  899    CTAATATTTTAAAGTATGTGTGCAAAACTTACTTCCCAGCCTCTAATAGAGAGGTTTACA  958

Seq_1  958    TGAAGGAGTTTTTGGTCACCAGGGTTAACACCTGGTTTTGTAAGTTTTCTAGGATAGATA  1017
              ||||||||||||| || || || || || |||||||||||||| || ||||| |||||||
Seq_2  959    TGAAGGAGTTTTTAGTAACTAGAGTAAATACCTGGTTTTGTAAATTCTCTAGAATAGATA  1018

Seq_1  1018   CTTTTTTATTATACAAGGGGGTAGCCCACAAAGGTGTAAATAGTGAGCAATTTTACAACG  1077
              ||||  |||| ||||| || ||||| |||||||||||| ||||||||||||||||||| |
Seq_2  1019   CTTTCCTATTGTACAAAGGTGTAGCGCACAAAGGTGTAGATAGTGAGCAATTTTACAAGG  1078

Seq_1  1078   CAATGGAAGATGCATGGCACTACAAAAAGACTCTTGCAATGTGTAACAGCGAGAGGATTC  1137
              |||||||||| |||||||| ||||||||||| || || ||||| ||||| ||||||||||
Seq_2  1079   CAATGGAAGACGCATGGCATTACAAAAAGACCCTCGCGATGTGCAACAGTGAGAGGATTC  1138

Seq_1  1138   TTCTTGAAGATTCCTCATCGGTCAATTACTGGTTCCCAAAAATGAGAGATATGGTCATAG  1197
              || | |||||||||||||| ||||| |||||||| ||||||||| | |||||||| || |
Seq_2  1139   TTTTAGAAGATTCCTCATCCGTCAACTACTGGTTTCCAAAAATGCGGGATATGGTGATCG  1198

Seq_1  1198   TTCCTCTATTCGACATATCTCTCGACACCAGTAAAAGGACCCGCAAAGAAGTCTTAGTGT  1257
              |||| || || ||||| | ||| || |||||||||||| | |||||||| ||||||||||
Seq_2  1199   TTCCGCTTTTTGACATTTGTCTTGAAACCAGTAAAAGGTCACGCAAAGAGGTCTTAGTGT  1258

Seq_1  1258   CAAAGGATTTTGTATTCACAGTTTTAAATCACATTCGCACTTATCAAGCCAAGGCACTTA  1317
              | ||||||||||| | |||||| |||||||| || || || || || || || || || |
Seq_2  1259   CCAAGGATTTTGTTTACACAGTGTTAAATCATATCCGTACATACCAGGCAAAAGCCCTGA  1318

Seq_1  1318   CATACTCCAATGTTTTATCCTTTGTCGAATCAATTCGTTCAAGGGTAATTATCAACGGAG  1377
              ||||| | |||||||||||||| ||||||||||||||||| ||||| || || || ||||
Seq_2  1319   CATACGCTAATGTTTTATCCTTCGTCGAATCAATTCGTTCGAGGGTGATCATTAATGGAG  1378

Seq_1  1378   TGACTGCCAGGTCTGAGTGGGATGTTGACAAATCTCTTTTGCAATCCTTGTCCATGACAT  1437
              | || || |||||||||||||||||||| |||||  | || |||||||| || ||||| |
Seq_2  1379   TCACCGCTAGGTCTGAGTGGGATGTTGATAAATCGTTATTACAATCCTTATCTATGACGT  1438

Seq_1  1438   TTTTCTTGCATACTAAGCTTGCCGTTTTAAAAGACGAATTGTTAATCAGCAAGTTTAGTT  1497
              ||||| | ||||| |||||| | ||  | |||||||| || || || ||||||||||||
Seq_2  1439   TTTTCCTACATACAAAGCTTTCTGTCCTTAAAGACGACTTATTGATAAGCAAGTTTAGCC  1498

Seq_1  1498   TGGGGCCAAAATCAGTAAGCCAGCATGTATGGGATGAGATTTCCCTGGCTTTTGGAAACG  1557
              | || || ||| ||||    ||   ||| ||||| ||||| ||  | || ||||| || |
Seq_2  1499   TTGGTCCCAAACCAGTGTCTCAATTTGTGTGGGAGGAGATATCATTAGCGTTTGGCAATG  1558

Seq_1  1558   CATTTCCATCGATCAAGGAGAGACTGCTAAATCGGAAACTAATTAAAGTGTCGGGAGACG  1617
              | |||||| | || ||||| ||| || | || || ||||| ||  || |  |||   | |
Seq_2  1559   CCTTTCCAACAATTAAGGAAAGATTGATCAACCGTAAACTGATCCAAATCACGGAGAATG  1618

Seq_1  1618   CATTAGAAATCAGGGTGCCTGATTTATATGTGACTTTCCACGATAGATTAGTGACTGAGT  1677
              | |||||||| ||||||||||||||||| || |||||||| ||||| || ||  | ||||
```

```
Seq_2  1619  CGTTAGAAATTAGGGTGCCTGATTTATACGTAACTTTCCATGATAGGTTGGTTTCCGAGT  1678

Seq_1  1678  ACAAAACATCGGTGGATATGCCAGTGCTTGATATCAGAAAGAGAATGGAGGAGACTGAGG  1737
             ||||    || || || ||||| | |||||||||||| |||| |||||| ||||| || |
Seq_2  1679  ACAAGCTCTCAGTCGAGATGCCGGCGCTTGATATCAGGAAGAAAATGGAAGAGACCGAAG  1738

Seq_1  1738  TTATGTACAATGCATTGTCTGAGCTATCTGTGCTCAAGGAGTCGGACAAGTTCGACGTTG  1797
             |||||||||||||| |||| || ||||| || || ||  | |||||||||||||| ||||
Seq_2  1739  TTATGTACAATGCACTGTCAGAACTATCCGTTCTTAAAAATTCGGACAAGTTCGATGTTG  1798

Seq_1  1798  ATGTTTTTTCCCGGATGTGCCAGACTTTGGAGGTAGACCCAATGACTGCAGCAAAGGTTA  1857
             ||||||||||||| ||||||||||  |||| || ||||| || |||||||||||| |||||||
Seq_2  1799  ATGTTTTTTCCCAGATGTGCCAATCTTTAGAAGTAGATCCTATGACTGCAGCGAAGGTTA  1858

Seq_1  1858  TTGTGGCAGTGATGAGCAACGAGAGCGGACTGACTCTTACATTCGAACAGCCAACTGAAG  1917
             ||||||| |||||||||||||||| || || |||||||| || || ||||| || ||||
Seq_2  1859  TTGTGGCCGTGATGAGCAACGAGAGTGGTCTTACTCTTACGTTTGAGCAGCCTACAGAAG  1918

Seq_1  1918  CAAATGTCGCATTGGCACTTAAAGATTCAGAAAAAGCCTCTGAGGGTGCACTAGTGGTTA  1977
             | ||| | ||| |||||||| |||| || |||||||| || || || ||  | || ||||
Seq_2  1919  CCAATATTGCACTGGCACTTCAAGACTCTGAAAAAGCTTCGGAAGGCGCGTTGGTAGTTA  1978

Seq_1  1978  CTTCTAGAGATGTTGAAGAACCATCCATGAAGGGTTCAATGGCAAGAGGAGAGTTACAAT  2037
             | ||  | ||||| || ||||| || ||||| |||||||||||  | || ||||||||||
Seq_2  1979  CCTCGCGTGATGTGGAGGAACCCTCTATGAAAGGTTCAATGGCCCGTGGTGAGTTACAAT  2038

Seq_1  2038  TGGCCGGTCT-GTCTGGAGACCAACCAGAGTCTTCCTATACTCGGAACGAGGAAATAGAG  2096
             |||||||  | | | || ||   ||||| ||||| | |||  ||| |||||| || |||
Seq_2  2039  TGGCCGGATTAG-CAGGTGACATTCCAGAATCTTCGTTTACAAGGAGCGAGGAGATTGAG  2097

Seq_1  2097  TCATTAGAGCAATTCCACATGGCAACGGCTAGTTCGTTAATTCGGAAACAGATGAGTTCG  2156
             || |||||||| || || |||||||| || |||||  | ||||  || |||||| |||||
Seq_2  2098  TCTTTAGAGCAGTTTCATATGGCAACAGCCAGTTCTCTGATTCAAAAGCAGATGTGTTCG  2157

Seq_1  2157  ATTGTGTACACGGGCCCCATTAAAGTTCAGCAAATGAAAAACTTTATTGATAGCCTGGTA  2216
             || |||||||| ||| |||||||||| || || ||||||||||||||||||||| ||||||
Seq_2  2158  ATCGTGTACACAGGCACCATTAAAGTACAACAGATGAAAAACTTTATTGATAGTCTGGTA  2217

Seq_1  2217  GCATCACTCTCTGCTGCGGTGTCGAACCTAGTCAAGATCCTAAAGGATACAGCTGCTATA  2276
             || || ||||||||||| || |||||| | || ||||||||| || ||||| |||||||| 
Seq_2  2218  GCCTCGCTCTCTGCTGCTGTTTCGAACTTGGTGAAGATCCTCAAAGATACTGCTGCTATT  2277

Seq_1  2277  GACCTCGAAACCCGTCAGAAGTTTGGAGTCTTAGATGTTGCGACCAAAAGATGGTTAATT  2336
             ||||| ||||| ||||||||||| |||||  | || ||| |  | || | |||| | |||
Seq_2  2278  GACCTTGAAACTCGTCAGAAGTTCGGAGTTCTGGACGTTACCTCAAAGAAATGGCTGATT  2337

Seq_1  2337  AAACCTTTAGCCAAGAATCACGCATGGGGCGTTATTGAAACACATGCTAGGAAGTACCAC  2396
             ||||| |  || ||||| || |||||||| ||| | ||||||||||||||||| ||||| 
Seq_2  2338  AAACCATCCGCAAAGAACCATGCATGGGGTGTTGTCGAAACACATGCTAGGAAATACCAT  2397

Seq_1  2397  GTTGCACTTTTGGAGTATGATGAGCATGGAGTGGTAACTTGCGACAGTTGGAGAAGGGTG  2456
             || |   |  ||||||||||||||  ||||||| | || ||||||  |||||||||||||
Seq_2  2398  GTCGTCTTGCTGGAGTATGATGAGTTTGGAGTGATCACGTGCGACGATTGGAGAAGGGTG  2457

Seq_1  2457  GCCGTGAGTTCTGAGTCAGTGGTTTATTCTGATATGGCAAAGCTCAGAACACTGAGGAGA  2516
             ||||| ||||||||| ||||| || || ||||||||||| ||||||| |||  ||||||  
Seq_2  2458  GCCGTAAGTTCTGAATCAGTAGTATACTCTGATATGGCCAAGCTCAAAACTTTGAGGAAG  2517
```

```
Seq_1  2517  TTATTAAGAGATGGTGAGCCTCATGTCAGCAGTGCTAAAGTCGTCCTAGTTGACGGTGTC  2576
             ||  |    ||||| || || |  || |||  |||||| || || || || ||||| || 
Seq_2  2518  TTGCTCTTGGATGGCGAACCACCGGTTAGCTCTGCTAAGGTTGTACTGGTAGACGGCGTT  2577

Seq_1  2577  CCGGGTTGTGGAAAGACAAAAGAGATTCTCTCGAAAGTAAATTTTGAGGAAGATCTAATC  2636
             ||||| || || ||||| || || ||||| ||||| ||||| ||||| ||||| || || 
Seq_2  2578  CCGGGATGCGGGAAGACGAAGGAAATTCTATCGAAGGTAAACTTTGATGAAGACCTGATT  2637

Seq_1  2637  TTAGTACCGGGTAAGCAGGCTGCTGAAATGATAAAGAGGCGTGCTAATGCGTCAGGAATA  2696
              | ||||| |||   || |||||||| ||||| |  ||  | || ||  |||| ||| ||
Seq_2  2638  CTCGTACCCGGTCGTCAAGCTGCTGAGATGATCAGAAGAAGAGCGAACTCGTCGGGAGTA  2697

Seq_1  2697  ATTCAAGCCACAAGAGATAATGTTCGTACTGTTGATTCATTTATAATGAATTACGGTAAA  2756
             ||   ||||||||  ||||||||  | || || ||||||||||| ||||||||||| || 
Seq_2  2698  ATAGTAGCCACAAAGGATAATGTCAGAACCGTCGATTCATTTATCATGAATTACGGGAAG  2757

Seq_1  2757  GGAACACGCTGTCAGTTCAAAAGGTTATTTATCGACGAAGGTCTGATGTTGCACACTGGT  2816
             |||   ||||| |||||||||||| | ||||| || |||||  ||||| |||||||||||
Seq_2  2758  GGAGGTCGCTGCCAGTTCAAAAGGCTTTTTATTGATGAAGGATTGATGCTGCACACTGGT  2817

Seq_1  2817  TGTGTGAATTTTCTTGTTTCTATGTCTCTGTGCGAAATTGCATATGTTTATGGAGACACA  2876
             |||||||||||  | ||  | |||||||||||||| ||||||||||| || |||||||| 
Seq_2  2818  TGTGTGAATTTCTTGGTAACAATGTCTCTGTGCGATATTGCATATGTATACGGAGACACC  2877

Seq_1  2877  CAACAAATTCCATACATCAACAGAGTATCCGGTTTTCCGTACCCTGCACATTTTGCAAAA  2936
             |||||||||||||| |||||||||||| |||| |||||||||||||| |||||| |||| 
Seq_2  2878  CAACAAATTCCATATATCAACAGAGTAACCGGATTTCCGTACCCTGCTCATTTTTCAAAG  2937

Seq_1  2937  ATAGAGGTTGATGAGGTGGAAACTCGCAGAACTACGCTGCGTTGTCCAGCCGACATTACC  2996
              | ||||| || |||||||| || ||||||||||||||||||||||| |||||  | |||
Seq_2  2938  CTGGAGGTGGACGAGGTGGAGACACGCAGAACTACGCTGCGTTGTCCGGCCGATGTCACC  2997

Seq_1  2997  CACTATCTTAACAGAAGGTACGAAGGACATGTCATGTGTACATCGTCGGTTAAAAAGTCA  3056
             || |   | ||    |||||||||||| |||| ||||| || ||  | |  || || || 
Seq_2  2998  CATTTCTTAAATCAGAGGTACGAAGGATATGTGATGTGCACTTCTGCAGAAAAGAAATCG  3057

Seq_1  3057  GTTTCTCAGGAAATGGTGAGCGGGGCCGCAATGATCAATCCTGTATCTAAGCCACTGAAT  3116
             || ||||| || ||||| || ||||| || |  |||||||| ||||| || ||||| | |
Seq_2  3058  GTATCTCAAGAGATGGTTAGTGGGGCTGCCACAATCAATCCAGTATCCAAACCACTCAGT  3117

Seq_1  3117  GGGAAAGTTTTGACTTTCACTCAGTCTGATAAAGAGGCGCTGCTTTCTCGAGGATATACG  3176
             || || || ||||| || || |||||||| || || ||| | ||  |||| ||| ||
Seq_2  3118  GGAAAGGTCTTGACCTTTACACAATCTGATAAGGAAGCACTGTTATCGAGAGGCTATTCG  3177

Seq_1  3177  GACGTCCATACAGTACATGAGGTACAAGGTGAGACATATGCAGATGTGTCGTTGGTCAGA  3236
             ||||||||||| || ||||| || ||||||||||||||||| ||||||||||| ||||||
Seq_2  3178  GACGTCCATACTGTGCATGAAGTTCAAGGTGAGACATATGCGGATGTGTCGTTAGTCAGA  3237

Seq_1  3237  TTGACTCCGACACCTGTATCTATCATCGCAGGAGATAGTCCACACGTTCTCGTAGCTTTG  3296
              ||||||||||||| ||||| ||||| ||| | || ||||| || || || || || |||
Seq_2  3238  CTGACTCCGACACCCGTATCCATCATTGCAAGGGACAGTCCCCATGTGCTTGTCGCATTG  3297

Seq_1  3297  TCAAGGCATACCCAAACATTGAAGTATTACACCGTAGTGATGGATCCTCTTGTAAGTATA  3356
             ||  |||| ||  || | || ||||| |||||||| || ||||||||| | || ||||||
Seq_2  3298  TCGCGGCACACAAAATCGTTCAAGTACTACACCGTTGTCATGGATCCTTTGGTTAGTATA  3357
```

```
Seq_1  3357  ATTAGGGATTTAGAAAAACTTAGTTCTTACTTGTTAGATATGTATAAAGTAGATGCAGGG  3416
             ||||| || |||||| |  |||||  |||  |  |||||||||| || || || |||||
Seq_2  3358  ATTAGAGAATTAGAACAGGTTAGTAGTTATCTCCTAGATATGTACAAGGTCGAGGCAGGT  3417

Seq_1  3417  ACCCAATAGCAATTACAGGTAGACTCCGTGTTTAAAGGTTCTAATCTTTTTGTTGCAGCA  3476
             || ||||||||||||||||||||||| ||||||||||||||||| ||||||||||| |||
Seq_2  3418  ACTCAATAGCAATTACAGGTAGACTCAGTGTTTAAAGGTTCTAACCTTTTTGTTGCGGCA  3477

Seq_1  3477  CCAAAGACTGGAGATATCTCAGATATGCAATTTTACTATGATAAGTGTCTCCCAGGTAAT  3536
             ||||||||||| || ||||| ||||||||||||||||||||||||||||| || || ||
Seq_2  3478  CCAAAGACTGGTGACATCTCTGATATGCAATTTTACTATGATAAGTGTCTTCCTGGGAAC  3537

Seq_1  3537  AGCACCATGTTAAATAACTATGATGCTGTTACCATGAGGTTGACTGACATTTCTCTTAAT  3596
             || || ||||| |||||||  |||||||||||||||| ||||||||||||||| ||||||
Seq_2  3538  AGTACGATGTTGAATAACTTCGATGCTGTTACCATGAAGTTGACTGACATTTCCCTTAAT  3597

Seq_1  3597  GTCAAAGATTGCATATTGGA-TTTCTCTAAGTCTGTGGCTGCACCGAAGGATCCGATCAA  3655
             ||||||||||||||||||||   | ||||||||||| || ||||||||||||  ||
Seq_2  3598  GTCAAAGATTGCATATTGGACA-TGTCTAAGTCTGTCGCAGCACCGAAGGATGAGAAAGT  3656

Seq_1  3656  ACCACTGATTCCAATGGTACGAACGGCGGCAGAAATGCCACGCCAGACTGGACTATTGGA  3715
               |  |||| ||||||||||||||||| ||||||||||||||||||||||||||||||||
Seq_2  3657  GACTTTGATACCAATGGTACGAACGGCTGCAGAAATGCCACGCCAGACTGGACTATTGGA  3716

Seq_1  3716  AAATTTGGTGGCGATGATCAAAAGAAACTTTAATTCACCGGAGTTATCAGGAATAATCGA  3775
             ||| || || || ||||||||||| |||||||||||||| ||||| || ||| || | ||
Seq_2  3717  AAACTTAGTTGCTATGATCAAAAGGAACTTTAATTCACCTGAGTTGTCCGGAGTAGTTGA  3776

Seq_1  3776  CATTGAGAATACTGCATCTTTAGTAGTAGATAAATTTTTTGATAGTTACTTGCTTAAAGA  3835
              |||||||| || || |||||||| ||||||||||||||||||||||| |||||||||||
Seq_2  3777  TATTGAGAACACAGCCTCTTTAGTGGTAGATAAATTTTTTGATAGTTATTTGCTTAAAGA  3836

Seq_1  3836  AAAAAGAAAACCAAATAAAAATGTTTCTTTATTTTGTAGAGAGTCTCTCAATAGATGGTT  3895
             ||||||||||||||| |||||| ||||||| ||| |||| |||||||||||||| |||||
Seq_2  3837  AAAAAGAAAACCAAACAAAAATTTTTCTTTGTTTAGTAGGGAGTCTCTCAATAGGTGGTT  3896

Seq_1  3896  AGAGAAGCAGGAGCAAGTGACCATTGGTCAGCTTGCAGATTTTGATTTTGTGGATCTTCC  3955
             ||  || || || ||||| || ||||| ||  |  | |||||||| ||||||||||| ||
Seq_2  3897  AGCCAAACAAGAACAAGTCACTATTGGGCAATTGTCTGATTTTGACTTTGTGGATCTGCC  3956

Seq_1  3956  TGCCGTTGATCAGTACAGGCATATGATTAAAGCGCAACCTAAGCAGAAGCTGGATACATC  4015
             ||| ||||||||||| || |||||||||||||| || || || |||||| ||||||| ||
Seq_2  3957  TGCAGTTGATCAGTATAGACATATGATTAAAGCACAGCCGAAACAGAAGTTGGATACTTC  4016

Seq_1  4016  AATTCAAAGCGAATATCCGGCCTTGCAGACGATTGTGTATCATTCGAAAAAGATCAACGC  4075
              |||||||  |||||||||||  | |||||||||||||||||||| || |||||||| ||
Seq_2  4017  CATTCAAACGGAATATCCGGCTCTACAGACGATTGTGTATCATTCTAAGAAGATCAATGC  4076

Seq_1  4076  AATCTTCGGTCCTTTGTTCAGTGAGCTCACAAGGCAAATGCTCGAAAGCATAGACTCAAG  4135
             ||| || || ||  | ||||| || || ||||||||| | || || ||  | ||||||||
Seq_2  4077  AATTTTTGGACCGCTCTTCAGCGAACTGACAAGGCAACTACTGGACAGTGTTGACTCAAG  4136

Seq_1  4136  TAAGTTTTTGTTCTTTACAAGGAAGACGCCAGCTCAAATTGAGGATTTCTTCGGAGATCT  4195
              |  ||||||||||| || |||||||| ||||||||||| || |||||||||||||||||
Seq_2  4137  CAGATTTTTGTTCTTCACGAGGAAGACACCAGCTCAAATCGAAGATTTCTTCGGAGATCT  4196
```

```
Seq_1  4196  CGATAGCCATGTCCCTATGGATATCTTGGAGTTGGATATTTCGAAGTATGACAAATCTCA  4255
              ||||| |||||||| |||||| | |||||||||||||||| || || |||||||||||
Seq_2  4197  AGATAGTCATGTCCCCATGGATGTGTTGGAGTTGGATATTTCCAAATACGACAAATCTCA  4256

Seq_1  4256  GAACGAGTTCCACTGTGCAGTAGAGTATGAAATATGGAGAAGACTTGGATTAGAAGATTT  4315
             ||| ||||| ||||| ||||||||||||||||| ||||||||||| ||  ||||||||||
Seq_2  4257  GAATGAGTTTCACTGCGCAGTAGAGTATGAAATCTGGAGAAGACTGGGTCTAGAAGATTT  4316

Seq_1  4316  TCTGGGAGAAGTTTGGAAACAAGGCCACAGGAAAACTACTCTTAAAGATTACACAGCTGG  4375
             ||||| |||||| ||||||||||| ||||| ||||| |||||||||||||||||||||||
Seq_2  4317  TCTGGCAGAAGTGTGGAAACAAGGGCACAGAAAAACCACTCTTAAAGATTACACAGCTGG  4376

Seq_1  4376  TATTAAAACGTGTTTATGGTACCAGAGAAAGAGTGGGGACGTTACAACATTCATCGGTAA  4435
             ||| ||||| |||||||||||||||||||||||||| || |||||||| ||||| || ||
Seq_2  4377  TATAAAAACATGTTTATGGTACCAGAGAAAGAGTGGTGATGTTACAACTTTCATAGGAAA  4436

Seq_1  4436  TACGGTGATTATTGCTGCTTGTTTAGCTTCCATGTTGCCCATGGAGAAAATAATCAAAGG  4495
             ||| || ||||| || || ||  |||| || ||||| || ||||| ||| | || |||||
Seq_2  4437  TACTGTTATTATAGCCGCGTGCCTAGCCTCGATGTTACCGATGGAAAAACTGATTAAAGG  4496

Seq_1  4496  TGCATTTTGCGGAGATGACAGTTTACTATACTTCCCAAAAGGTTGTGAGTTTCCTGACAT  4555
              || ||||| || ||||||||| | || ||||||||||| || |||||||| ||||| ||
Seq_2  4497  GGCGTTTTGTGGTGATGACAGTCTGCTGTACTTCCCAAAGGGCTGTGAGTTCCCTGATAT  4556

Seq_1  4556  ACAGCATACAGCCAACCTTATGTGGAATTTCGAGGCTAAGCTATTCAGAAAGCAGTATGG  4615
             ||| ||   | || ||| | |||||||| || || || ||| | | ||  ||  |||| ||
Seq_2  4557  ACAACAGGCTGCAAACTTAATGTGGAACTTTGAAGCCAAGTTGTACAAGAAAAAGTACGG  4616

Seq_1  4616  TTATTTCTGTGGAAGGTACGTGATACATCATGACAGAGGGTGTATTGTTTATTATGACCC  4675
              || |||||||||||||||||||||||||||| ||||| ||||| || |||||||| ||
Seq_2  4617  GTACTTCTGTGGAAGGTACGTGATACATCATGATAGAGGTTGTATAGTATATTATGATCC  4676

Seq_1  4676  TTTGAAGTTGATTTCTAAACTTGGTGCTAAACACATCAAGGATTGGGATCACTTAGAAGA  4735
             | | |||||||| || ||||| ||||| |||||||||||||||||||||||  |||||||
Seq_2  4677  TCTTAAGTTGATCTCAAAACTCGGTGCAAAACACATCAAGGATTGGGATCATCTAGAAGA  4736

Seq_1  4736  GTTCAGAAGATCCCTTTGTGATGTTGCAAATTCGTTGAACAACTGTGCGTATTACACGCA  4795
             ||||||||||||||| |||||||||||    ||||||||||| ||||||||||||| |||
Seq_2  4737  GTTCAGAAGATCCCTCTGTGATGTTGCTGGCTCGTTGAACAATTGTGCGTATTACATGCA  4796

Seq_1  4796  GTTGGACGACGCTGTGAGTGAGGTCCATAAAACCGCACCCCCGGGTTCGTTTGTGTATAA  4855
              ||||||||||||||  | ||||| ||||||||||| ||||| ||||||||||| |||||
Seq_2  4797  ATTGGACGACGCTGTTGGGGAGGTTCATAAAACCGCCCCCCCTGGTTCGTTTGTTTATAA  4856

Seq_1  4856  AAGTTTAGTTAAATATCTGTCCGATAAGGTTCTTTTTAGAAGTTTGTTTATAGAT-GG-C  4913
             ||||||||||||||||||||| |||||||||| | ||||||||||||||||  || |  |
Seq_2  4857  AAGTTTAGTTAAATATCTGTCAGATAAGGTTTTGTTTAGAAGTTTGTTTATCAATGGCTC  4916

Seq_1  4914  T-CTTGTTAAGGGTAAAGTCAATATTAATGAGTTCATAGACTTGTCAAAATCAGAAAAAT  4972
             |   |||||  ||||||||  |  ||||  ||||| || |||||||| || |||||||  |
Seq_2  4917  TAACTGTTAGTGGTAAAGTTAGAATTAGCGAGTTTATCGACTTGTCTAAGTCAGAAAGGT  4976

Seq_1  4973  TTCTTCCGTCTATGTTCACACCTGTTAAGAGTGTCATGATCTCCAAGGTTGATAAGATAT  5032
             | || |||||||||||||| | |||||| || |||    |||| |||||||| ||| |
Seq_2  4977  TGCTGCCGTCTATGTTCACTCATGTTAAAAGCGTCTCTGTCTCAAAGGTTGACAAGGTCA  5036

Seq_1  5033  TGGTTCATGAAGATGAATCTTTGTCCGAAGTCAATTTACTCAAAGGTGTAAAACTCATTG  5092
```

```
             ||||| ||||||| || ||||| || |||||||| ||  | || || || ||||| || |
Seq_2  5037  TGGTTAATGAAGAAGATTCTTTATCAGAAGTCAACTTGTTGAAGGGCGTTAAACTTATAG  5096

Seq_1  5093  ATGGTGGCTATGTACATCTTGCTGGTCTTGTGGTGACAGGTGAATGGAATTTGCCAGATA  5152
             ||||||| || ||   ||| |||||||| || ||| | ||||| |||||| | ||||| |
Seq_2  5097  ATGGTGGTTACGTTTGTCTGGCTGGTCTAGTAGTGTCCGGTGAGTGGAATCTTCCAGACA  5156

Seq_1  5153  ATTGTCGTGGTGGTGTCAGTGTCTGTTTGGTCGATAAGAGAATGGAGAGAGCGGACGAGG  5212
             |||||||||||||||||||  |||| ||||||||||| |||||| | |||||||| || |
Seq_2  5157  ATTGTCGTGGTGGTGTCAGCATCTGCTTGGTCGATAAAAGAATGCAAAGAGCGGATGAAG  5216

Seq_1  5213  CAACTCTTGCTTCATACTATACCGCAGCGGCTAAGAAAAGGTTTCAGTTCAAAGTCGTTC  5272
             | || ||||  || || ||||| | ||| || ||||||||||| ||||| ||  ||||||
Seq_2  5217  CGACACTTGGATCGTATTATACTGGAGCTGCAAAGAAAAGGTTCCAGTTTAAGATCGTTC  5276

Seq_1  5273  CAAATTACAACATCACTACCAAGGACGCAGAAAAGGCAGTTTGGCAAGTACTAGTTAATA  5332
             |||| |||    || || || ||||| || |||| |    | |||||||| ||||||||||
Seq_2  5277  CAAACTACGCAATTACAACTAAGGATGCGGAAAGGAACATATGGCAAGTCCTAGTTAATA  5336

Seq_1  5333  TTAGAAATGTTAAAATTGCTGCGGGTTACTGTCCGCTGTCATTAGAATTTGTGTCAGTGT  5392
             |||| ||||| || || ||||| ||||| |||||| ||||| |||||||||||||| ||||
Seq_2  5337  TTAGGAATGTCAAGATGGCTGGGGGTTTCTGTCCCCTGTCGTTAGAATTTGTGTCTGTGT  5396

Seq_1  5393  GTATTGTTTATAAAAATATTATAAAACTCGGTTTGAGAGAGAAAATTACGAGCGTCACGG  5452
             |||| ||||||||||||| ||||||| | |||||||| |||| ||||| || ||    |
Seq_2  5397  GTATAGTTTATAAAAATAATATAAAATTGGGTTTGAGGGAGAAGATTACAAGAGTGGATG  5456

Seq_1  5453  ATGGAGGGCCCATGGAACTATCAGAAGAAGTTGTTGATGAGTTCATGGA-AGAA-GTCCC  5510
             | | ||| ||||| |||||  | |||||||||||||||||||||||||| ||   | |
Seq_2  5457  ACGCAGGTCCCATTGAACTTACCGAAGAAGTTGTTGATGAGTTCATGGAGAGTGTGCCTA  5516

Seq_1  5511  GAT-GTCTGTAAGGCTTGCAAAATTTCGTTCGAAGACCGGAAAAAAGTTTAGTAGTAAAA  5569
               |             |  |                      |   || |      |    ||| |
Seq_2  5517  TGTCAGTCAGGCTTGCTAAATTTCGAACCAAATCCTCAAAAAGAGGTCCGAAACATAATA  5576

Seq_1  5570  GTGAGAATAATAGTGGTAATAATAGGCCGAAACCAAACAAAAACCAAAGGAAGGAAAAGG  5629
             || | |||| || ||  |  ||  ||| |        |    ||  ||    |  |  ||
Seq_2  5577  GTAATAATACTAATGAAAGAAAAGGGCGGTCTAATTTCCGTAAGAAACAAGACCAGGAGA  5636

Seq_1  5630  GTTTAAAAGTTAGGGTTGAGAAGGATAATTTAATTGATAATGAATTGGAGACTTACATCG  5689
             |||    |||||| | |      |||||||| |||||  ||||    |||||| |   |||
Seq_2  5637  GTTATGGAGTTAGTGATAGTTTAGATAATTTGATTGAAGATGATACCGAGACGTCAGTCG  5696

Seq_1  5690  CCGATTCAGATTCGTATTAAATATGTCTTACACAATCGCAACTCCATCGCAATTTGTGTT  5749
             | |  || |||||||||||||||||||||| | | ||  |  |||| || ||||| |||||
Seq_2  5697  CGGGATCTGATTCGTATTAAATATGTCTTACGCTATTACTTCTCCGTCACAATTCGTGTT  5756

Seq_1  5750  TTTGTCATCAGCATGGGCCGACCCTATAGAATTAATAAATTTATGTACTAATTCACTAGG  5809
             ||||||||||||||||||||||||| |||||||||||||||| | |||||||||||  ||||
Seq_2  5757  TTTGTCATCAGCATGGGCCGACCCTGTAGAATTAATAAATATTTGTACTAATTCGTTAGG  5816

Seq_1  5810  TAATCAGTTCCAAACACAACAAGCTAGAACAACCGTTCAACGGCAATTTAGCGAAGTGTG  5869
             ||| ||||| |||||||||||||||| || || || |||||| ||| || ||||| |||||
Seq_2  5817  TAACCAGTTTCAAACACAACAAGCAAGGACTACTGTTCAACAGCAGTTCAGCGAGGTGTG  5876

Seq_1  5870  GAAACCTGTCCCTCAAGTCACTGTTAGGTTTCCTGACAGTGGTTTTAAGGTGTATAGGTA  5929
             ||||||| ||||||||   ||||| ||||| ||||||| ||  |||||||||||||||||
```

```
Seq_2  5877  GAAACCTTTCCCTCAAAGTACTGTCAGGTTCCCTGACAATGTATTTAAGGTGTATAGGTA  5936

Seq_1  5930  CAATGCGGTACTAGATCCTCTAGTTACTGCTTTGTTAGGAGCTTTCGATACTAGAAATAG  5989
              ||||||||  ||||||||||| ||||||| ||| | ||| |||||||||||||||||||
Seq_2  5937  TAATGCGGTTATAGATCCTCTAATTACTGCATTGCTGGGAACTTTCGATACTAGAAATAG  5996

Seq_1  5990  GATTATAGAAGTCGAAAATCAGGCGAACCCGACAACCGCCGAAACGTTAGACGCTACTCG  6049
              | ||    ||    |||    || || ||      |  |  ||   | |
Seq_2  5997  -AATAATAGAGGTAGAAAATCAGCAAAGCCCGACTACAGCCGAAACATTGGATGCCACTC  6055

Seq_1  6050  TAGAGTAGATGACGCAACGGTGGCTATAAGGAGCGCTATAAATAATTTAGTAGTAGAATT  6109
                    |           |          |  |       |  | ||      |  | |
Seq_2  6056  GCAGAGTGGACGATGCTACGGTTGCGATCAGGTCCGCTGTTAATAATTTAGTTAATGAAT  6115

Seq_1  6110  GGTCAAAGGAACAGGTTTGTACAATCAGAGCACATTTGAAAGTGCATCCGGTTTACAATG  6169
              |  ||  | |   | ||     | |        |||  ||       | |  |
Seq_2  6116  TGGTAAGAGGAACAGGTTTCTACAACCAGAGTACTTTCGAAAGTATGTCTGGGTTGGCCT  6175

Seq_1  6170  GTCCTCTGCACCTGCATCTTGAGATAATCGAGATGCTTAAATAACAGATTGTGTCTGCAA  6229
             |  ||      |         |  ||||  ||      |   ||  | |||  ||   ||
Seq_2  6176  GGACTTCTGCGCCAGCGTCCTAAGTAATAAAGGACGAAATTAAAGGAAGTGTATCCTAAA  6235

Seq_1  6230  ACACACGTGGTACGTACGATAACGTATAGTGTTTTTCCCTCCACTTAAATCGAAGGGTAG  6289
             | ||||||||| |||||||||||||| |||||||||||||||||||||||||||||||
Seq_2  6236  ATACACGTGGTGCGTACGATAACGTACAGTGTTTTTCCCTCCACTTAAATCGAAGGGT-T  6294

Seq_1  6290  -TGTCTTGGAGCGCGCGGGACAAATGTGTATGGTTCATACACATCCGTAGGCACGTAATA  6348
              ||||||||| ||||||||  |||| |  |||||||||  | |||||||| || ||||||
Seq_2  6295  TTGTCTTGGAACGCGCGGGTTAAATATACATGGTTCATGTATATCCGTAGACAAGTAATA  6354

Seq_1  6349  AAGCGAGGGATTCGAATTCCCCCGGAACCCCCGGTAGGGGCCCA  6392
             | ||| ||||||||||||||||||||||||||||||||||||||
Seq_2  6355  ATGCGTGGGATTCGAATTCCCCCGGAACCCCCGGTAGGGGCCCA  6398
```

Features [Seq_1]:

```
RP1   : [972 : 991]
RP2   : [1999 : 2019]
RP3   : [2965 : 2984]
RP4   : [3990 : 4009]
RP5   : [5182 : 5201]
RP6   : [6373 : 6392]
```
