# Supplementary material for: Nanopore Technology Applied to Targeted Detection of Tomato Brown Rugose Fruit Virus Allows Sequencing of Related Viruses and the Diagnosis of Mixed Infections
Source: Plants (Basel). 2023 Feb 22;12(5):999. doi: 10.3390/plants12050999 (PMC10005216; doi:10.3390/plants12050999)
Supplement: Supplementary file 1 [file plants-12-00999-s001.zip › plants-2203162-Table S1.pdf]

Supplemental Table S1. List of primers used in this study, and their nucleotide sequences

**Primer name**	**Primer sequence (5' > 3')**
Reverse primers for cDNA ONT sequencing	
ToBRFV_RP1_AP+*adapter*	*ACTTGCCTGTCGCTCTATCTTC*CAGGTGTTAACCCTGGTGAC
ToBRFV_RP2_AP+*adapter*	*ACTTGCCTGTCGCTCTATCTTC*CCATTGAACCCTTCATGGATG
ToBRFV_RP3_AP+*adapter*	*ACTTGCCTGTCGCTCTATCTTC*TGGACAACGCAGCGTAGTTC
ToBRFV_RP4_AP+*adapter*	*ACTTGCCTGTCGCTCTATCTTC*TCCAGCTTCTGCTTAGGTTG
ToBRFV_RP5_AP+*adapter*	*ACTTGCCTGTCGCTCTATCTTC*CTCTCCATTCTCTTATCGAC
ToBRFV_RP6_AP+*adapter*	*ACTTGCCTGTCGCTCTATCTTC*TGGGCCCCTACCGGGGGTTC
RT-PCR (Alkowni et al., 2019)	
ToBRFV-F	AATGTCCATGTTTGTTACGCC
ToBRFV-R	CGAATGTGATTTAAAACTGTGAAT
RT-qPCR (EPPO Bullettin, 2021)	
CaTa28 Fw	GGTGGTGTCAGTGTCTGTTT
CaTa28 Rv	GCGTCCTTGGTAGTGATGTT
CaTa28 Pr	5’-6FAM-AGAGAATGGAGAGAGCGGACGAGG-BHQ’1-3’
CSP1325 Fw	CATTTGAAAGTGCATCCGGTTT
CSP1325 Rv	GTACCACGTGTGTTTGCAGACA
CSP1325 Pr	5’-Cy5-ATGGTCCTCTGCACCTGCATCTTGAGA-BHQ’1-3’
RT-PCR for Sanger sequencing of 5’- and 3’- terminal regions	
ToBRFV-3’-for	TGGTGGTGTCAGTGTCTGTTT
ToBRFV-3’-rev	TGGGCCCCTACCGGGGGTT
PepMV-5’-for	GAAAACAAAACATAACACATAATAT
PepMV-5’-rev	GGCTTTGTGGCATTAACATG
PepMV-3’-for	AACCAACCTACAGCTTCTAAC
PepMV-3’-rev	TTGTTTAGTAGATTTAGATACTAAG
