# Supplementary figures and images for: Nanopore Technology Applied to Targeted Detection of Tomato Brown Rugose Fruit Virus Allows Sequencing of Related Viruses and the Diagnosis of Mixed Infections
Source: Plants (Basel). 2023 Feb 22;12(5):999. doi: 10.3390/plants12050999 (PMC10005216; doi:10.3390/plants12050999)

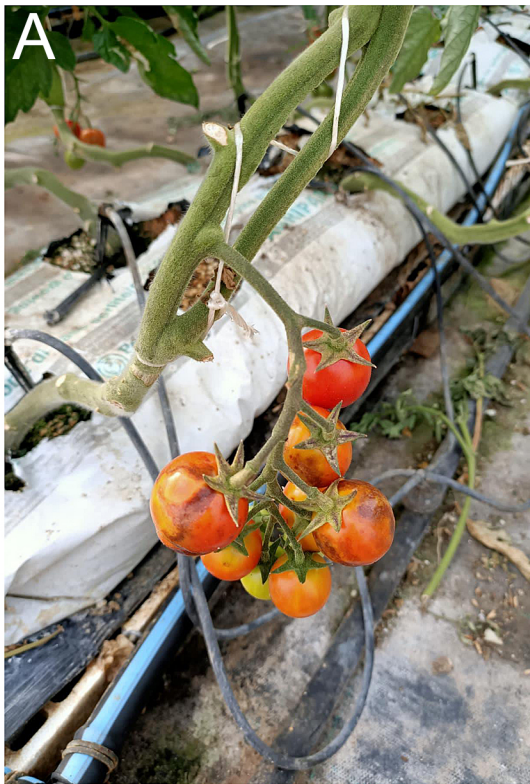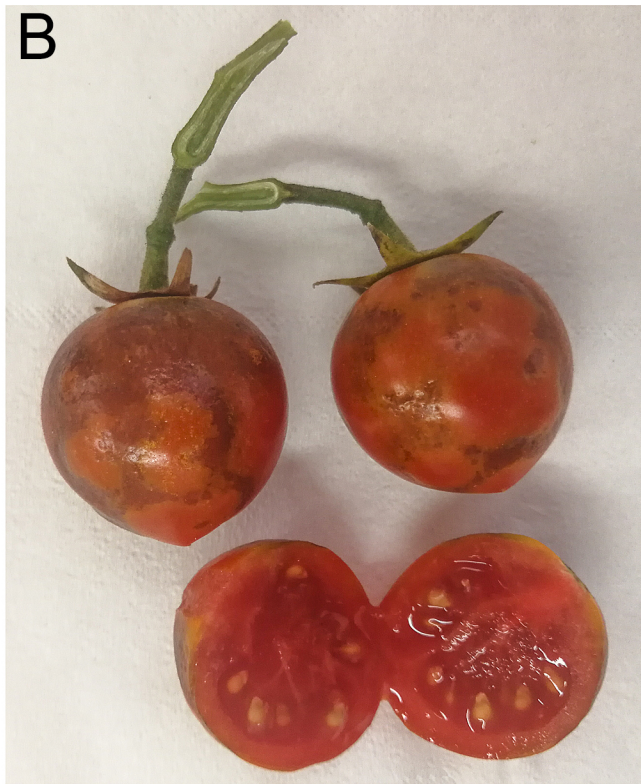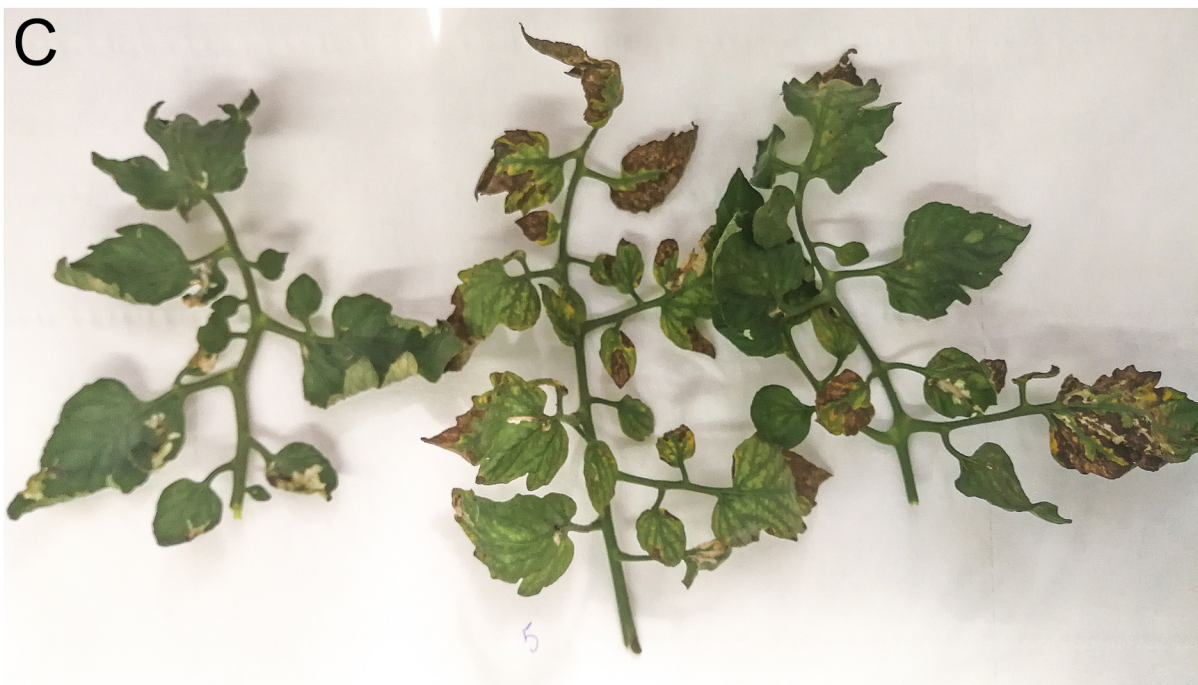

Supplement: Supplementary file 1 [file plants-12-00999-s001.zip › plants-2203162-Figure S1.pdf]
